# The Cholera

**Published:** 1849-01

**Authors:** 


### EDITORIAL DEPARTMENT.

*The Cholera.*—The question, "will the Cholera revisit this continent?" so often, for several months past, in the mouths of physicians and others, is at last settled by the recent appearance of this dreaded Epidemic at New York. That it will appear at other places, and extend itself, to a greater or less extent, over the country, is to be expected from the history of its former visitation, and the course which it has pursued in other countries. The painful responsibility of treating the disease may at any moment fall to the lot of any one of our medical readers, wherever situated.

Are we prepared to meet the emergency by settled, rational views of its character and management? What is the actual state of our present knowledge of its causes, pathology and therapeutics? These questions must arise in the mind of every Medical Practitioner, and claim his earnest, candid consideration. We feel that we need offer no apology for submitting, at this juncture, a few reflections upon the subject, although well aware that many of our readers have greatly the advantage of us, both as regards practical acquaintance with the disease, and familiarity with its literature. While we would urge upon them the duty of communicating freely the conclusions at which they may have arrived by observation, reflection and study, tendering the pages of this Journal for that purpose, we will venture a few considerations, which we offer chiefly as suggestions, to call out better and more extended discussion by some of our correspondents, whose opinions are entitled to more weight than those which may be uttered by us.

Directing our attention, at once, to the subject in a practical point of view, we are led to consider whether at this moment our knowledge is farther advanced than at the previous prevalence of this epidemic, and if so, in what particulars our acquaintance with it may be regarded more complete and satisfactory? It must be obvious that, as a point of departure for a fair examination of the subject, it is important to survey the amount of real information which science may be considered to have acquired, defining, as clearly as practicable, the boundary lines separating the known from the unknown, and not failing to estimate correctly the latter, as well as the former. Of the assemblage of phenomena characterizing the affection, and of its diagnosis, we need say nothing, for, under these aspects, the disease is sufficiently familiar. Without any enumeration of symptoms, then, we proceed to inquire concerning their relationships and dependencies.

The first prominent event in the clinical history is the evacuation of serous or sero-fibrinous fluid from the stomach and intestines. This is the first link in the chain of *appreciable* phenomena belonging to the disease proper. This, in the majority of cases, it is true, is preceded by more or less diarrhoea of a common character. The latter ushers in the disease, constituting the premonitory symptoms, but do not, strictly speaking, constitute a portion of the disease. That is to say, true Cholera, as a distinctive affection, does not commence until the serous discharges are apparent. Notwithstanding some reported analyses of the choleric evacuations which have lately been reported, it may probably be assumed as satisfactorily established, that these characteristic discharges consist of the serum of the blood, commingled, generally, with fibrin or lymph—in other words they consist of the *liquor sanguinis*. The blood thus loses more or less of its important constituents—constituents essential to its circulation, the changes it should undergo in the lungs and elsewhere, and to the several purposes which it is designed to fulfil in the economy. We suppose it to be pretty conclusively settled, that most of the phenomena which subsequently occur in the progress of the disease, are dependent upon this primary event. Certainly the series of distinctive symptoms which successively develop themselves, are capable of being rationally explained by reference to each other, and to the loss of the liquor sanguinis as the first appreciable proximate cause. The enfeebled circulation and pulselessness; the defective calorification and hæmatosis, giving rise to the extreme coldness of the surface and of the breath, the lividity amounting to cyanosis, and even asphyxia, and the unchanged atmosphere returned by expiration; the spasms; the cessation of transpiration and secreted fluids; the urgent thirst, &c., all which are the distinguishing external features of the disease, are clearly enough explicable by reference to the correlation of functions, and the impaired constitution of the blood. The latter is the first step in the series, and the former are sequences legitimately deducible from it. All these results may be intelligibly associated together, and collectively traced to the source just mentioned, but this the reader will readily do for himself.

What, then, the inquiry next arises, occasions the effusion of the serum of the blood into the intestinal tube? This question, if we could answer it, would bring us to the essential morbid condition in which the affection consists, or, in other words, to the true proximate cause of the disease. The answer to this question involves full knowledge of the morbid state, or states, anterior to the serous evacuations, upon which the latter are dependent, and, also, full knowledge of the external or remote cause of the disease. Do we possess any information on these points? We must reply in the negative. Recollecting that we are always on safest ground, and nearer the truth, when we do not assume to have knowledge to which we have no valid claims, let us freely confess our utter ignorance of the essential nature and the ætiology of cholera. Investigation has shed no inconsiderable amount of light on the pathological history of the disease. But has not, as yet, carried us beyond sequences. This, alas! is by no means a single instance of like imperfection of our knowledge, and that we are peculiarly alive to the poverty of our science, in this instance, is owing to the enormous fataility of the disease, which leads us to experience more keenly the desire to bring it under the control of art.

Reflection upon the possible sources of that inappreciable and unknown morbid influence which produces the first appreciable and known clinical event in the history of the disease, will, as we think, lead to the conclusion that it is to be referred to the following, singly, or combined, to wit:

1. Perversion or poisoning of the blood.
2. A local morbid condition of the intestinal mucus surface.
3. Disorder of the nervous system.

To each of these, the proximate causation of the disease has been assigned by different writers. That the essential pathology lies within the compass of the three hypotheses is altogether probable, because we cannot conceive of the production of the morbid phenomena which characterize the disease except as emanating from one or more of these three departmentsof the organism. To discuss the respective merits of these hypotheses would require not a little time and space, and does not fall within our present purpose. Our own opinion (which we give for what it is worth) is in favor of the humoral doctrine. It seems to us most rational to suppose that the immediate action of the remote cause (whatever it be) is upon the blood: that a morbific principle pervades the atmosphere, which either vitiates the circulating fluid, or excites a fluxion to the gastro-intestinal mucous membrane, in order for its elimination, carrying with it the important elements of the blood itself, which appear in the discharges peculiar to the disease. We base this opinion, not upon positive facts, for the opinion is, in the present state of knowledge, purely hypothetical; it is not pretended that a morbid change in the blood, or a morbid principle in it, have been ascertained. But the supposition comports best with the known circumstances involved in the production of the disease, and in its history, and also with its analogies to other general diseases, more especially the essential fevers, with which, by some writers, cholera has been classed. The opinion, moreover, is strengthened by the absence of positive evidencesof constant, adequate lesions either of the intestinal mucous membrane, or nervous system, which we have more right to assume should be discoverable if the disease were due to one or both of these sources. With our present knowledge of the general pathology of the blood, it is not strange that its morbid condition in cholera should be of an occult nature, for this is the case in other instances in which the existence of unknown morbid conditions of the blood standing in a causative relation to diseases is abundantly established; but if the seat were in the nervous system, or intestinal membrane, (more especially in the latter,) we should much more expect that pathological appearances should have been discovered, and the fact that no uniform lesions have been ascertained, is much stronger evidence against the hypotheses which refer the disease to solid structures, than the same absence of positive knowledge is evidence against the existence of humoral changes which our means of analysis do not, as yet, enable us to discern.

Leaving theoretical reflections, and reverting to the question, has our actual knowledge of cholera advanced, and, if so, in what particulars, we are bound, as it seems to us, to acknowledge, that, as regards its pathology, all that may be considered to have been elucidated relates to the mutual relations of its secondary phenomena, the seat and nature of the prime, proximate cause being now, as heretofore, wholly undemonstrated, and a fair subject for differences of speculative opinion.

The aetiology almost necessarily occupies the same position with the pathology, both doubtless, being so closely connected that our knowledge of both must advance *pari passu.* The remote cause must be of a specific nature, for the disease not only possesses, in a striking degree, specific characters, but presents, in a remarkable manner, uniformity as respects these characters in all quarters of the globe. Of the diseases ordinarily attributed to specific agencies there are few which do not manifest greater variations in particular situations, and in individual cases. The cause is not only specific, but it is not easily affected by ordinary, modifying influences.

Equally certain is it, that the cause is epidemic. The supposition that an endemial specific cause is generated in each country, and portion of country in which the disease successively appears, is rendered absurd by the law of probabilities. Such a series of coincidencies as this supposition involves, is inconceivable. Our knowledge of the laws and conditions regulating the diffusion of the epidemic cause, is very meager. That it resides in the atmosphere is altogether probable, but in its transportation from one place to another, it does not appear to conform to atmospheric currents. The only positive evidence bearing upon the subject of its existence in the atmosphere that has been communicated, (so far as our knowledge extends) is that afforded by the observations of Dr. Prout, in the epidemic of 1832. Dr. P. observed a distinct barometrical change, denoting increased weight of the atmosphere, continuing steadily during the prevalence of the disease in London, which, as he thought, denoted the presence of a ponderous gas. But we are not aware that this has been confirmed by similar results in other places and periods; nor do we hear any confirmation, by the same observer, during the recent re-appearance of the epidemic in England. The progress of the disease shows a greater liability to be attacked by it among the inhabitants of towns, and unhealthy situations, than among those who reside in salubrious country regions. This, however, does not prove that the remote cause is more abundant in places where the disease most prevails, but that subsidiary morbid influences are more rife in those places, giving to the specific miasm greater efficiency, and rendering the system less able to resist its influences. That this is the true explanation, is shown by the fact that, of the population of a place where the disease prevails, the great proportion of its victims are among the classes who, on account of situation, habits of life, fear of the disease, occupation, state of health, &c., are more exposed to collateral morbid agencies, while they do not probably imbibe more of the poisoned atmosphere than those, in the same places, who are exempt from the disease. An atmospherical condition, capable, under favorable circumstances, of inducing cholera, it is most reasonable to think, must be widely diffused at periods when the disease prevails. This condition is indicated in places where cholera does not prevail, by an unusual proclivity to ordinary diarrhœa. This fact was noticed in this country during the previous visitation of this epidemic; and, in so far as we may judge from the limited sphere of our own observations, a state approaching to such a condition exists in this place at the time we are writing. We have certainly known and heard of an unusual number of cases of diarrhœa, of late, for the present season of the year, and we should not be surprised if this were the case generally throughout the country. If what we have noticed has any significancy as a forerunner of the epidemic, an increased accumulation in the atmosphere of the specific cause, and the co-operation of other morbid causes, will be likely to determine, ere long, the occurrence of cases of cholera. The relative agency of subsidiary causes in the ætiology has an important practical bearing upon the prophy laxis which is too obvious to require comment. As regards the question of contagion, the preponderance of medical opinion is greatly for the negative, yet the affirmative has its adherents in the medical profession, and the doctrine of its *contingent contagiousness* is entertained by some writers of eminence. Some circumstances, upon superficial examination, appear to sustain the popular tendency to view the disease as contagious—for example, its apparent transportation in vessels from Germany to Great Britain, in the epidemic now existing in the latter country; the first appearance of the disease at the Quarantine, near New York; its migration along thoroughfares, etc. These circumstances, are, however, it is believed, satisfactorily explained, irrespective of any admission of a contagious principle; and they are opposed by facts of a more weighty character which conflict with such an admission. We have accounts of the greatest possible degree of exposure to a contagious miasm, (if it exists,) experiments of inoculation, &c., with negative results. Nor do we find, in places where the disease prevails, that of a given number of persons attending upon the sick, a larger ratio are attacked, than of the same number which may be selected in the same places who do not come into contact with the disease. The disease often appears in places and families completely isolated from any contagious influence, nor is it a general rule, that when cases occur, other cases follow in the same vicinity, in such a manner as to denote that the former occasioned a new focus for the extension of the disease. To these considerations, it may be added, that the character and progress of the disease are not favorable (judged by analogy) for the development of a poisonous emanation. Its rapid career, and the fact that its phenomena indicate diminution and abolition of functions, rather than vitiation, are opposed to the idea of contagion.

Candid examination of the merits of this important question, must, we think, satisfy every unbiassed mind, that the small evidence in the affirmative is overbalanced by an amount of evidence for the negative, which is nearly, if not altogether, adequate to establish the latter position.*

---

* The supposition that, in general, the diffusion of the disease does not involve a contagious principle, but that under certain circumstances, as yet not well understood, a contagious miasm may be generated, will perhaps best explain all the facts bearing on this point. This view is maintained by Prof. C. A. Lee, in his notes to the article on Choleric Pestilence, in Copland's Medical Dictionary, to which the reader is referred for a comprehensive and able discussion of the subject.

And of the causation of the disease in general, it may be remarked, that one of the best illustrations of our absolute poverty of knowledge on the subject, is afforded by the fact, that a hypothesis, as purely gratuitous as can well be imagined, after all, is adequate to explain the progress and diffusion of the disease, as well, if not better than others; we allude to the fanciful doctrine of the animalcular origin of cholera, maintained by Dr. Holland and others.

In the foregoing remarks, we have endeavored to sketch briefly the knowledge which medical science, in the present stage of its progress, possesses, of the pathology and causes of epidemic cholera. It is quite needless to disclaim any pretensions to elaborateness in what we have written. For this we have neither time nor space, saying nothing of resources and ability. This is not wanted. The literature of cholera is already sufficiently copious; in fact, so much so, as rather to repel, than invite investigation of the subject. But in the view of probability that we we may soon be called upon to treat the disease, it behoves the medical practitioner to be prepared for this responsibility. And this preparation, as it appears to us, involves, first of all, a clear apprehension of what *is* known, and what is *not* known, respecting its character and origin. We may then proceed to arrive at some definite views, relating more immediately to its prevention and treatment. The latter topics we reserve for another number.

If the views which we have presented be correct, our knowledge of cholera certainly falls very far short of what could be desired; but the question which presses upon us, and which should regulate our practical conduct in this, as in all other points, is not, what could we wish to know, but what do we actually know. And it is a consideration which should never be lost sight of, that, in medicine, a just appreciation of the limits of our knowledge—of its poverty and imperfections—has practical relations of not less importance, than an equally just appreciation of all that science and art have discovered and rendered available. True philosophy and humanity dictate, not to estimate the value of what we have, by an imaginary sense of what is unattainable, or yet to be attained, but to make the best use of present resources, hoping and striving constantly for their continued accumulation.

